# NSCLC中TRAF家族蛋白的表达和预后以及功能综合分析

**DOI:** 10.3779/j.issn.1009-3419.2025.102.09

**Published:** 2025-03-20

**Authors:** Yixuan WANG, Qiang CHEN, Yaguang FAN, Shuqi TU, Yang ZHANG, Xiuwen ZHANG, Hongli PAN, Xuexia ZHOU, Xuebing LI

**Affiliations:** ^1^300052 天津，天津医科大学总医院，天津市肺癌研究所（王逸璇，陈嫱，范亚光，涂姝祺，张洋，张修闻，潘红丽，李雪冰）; ^1^Tianjin Key Laboratory of Lung Cancer Metastasis and Tumor Microenvironment, Tianjin Lung Cancer Institute, Tianjin Medical University General Hospital, Tianjin 300052, China; ^2^300222 天津，天津市胸科医院呼吸与危重症医学科（陈嫱）; ^2^Department of Respiratory and Critical Medicine, Tianjin Chest Hospital, Tianjin 300222, China; ^3^300052 天津，天津医科大学总医院，天津市神经病学研究所（周雪霞）; ^3^Tianjin Key Laboratory of Injuries, Variations and Regeneration of the Nervous System, Tianjin Neurological Institute, Tianjin Medical University General Hospital, Tianjin 300052, China

**Keywords:** 肺肿瘤, 肿瘤坏死因子, 肿瘤坏死因子受体相关因子, 预后, 功能, Lung neoplasms, Tumor necrosis factor, Tumor necrosis factor receptor associated factor, Prognosis, Function

## Abstract

**背景与目的:**

肺癌是目前世界上发病率和死亡率较高的恶性肿瘤之一。然而，肺癌进展的确切机制仍不清楚。肿瘤坏死因子受体相关因子（tumor necrosis factor receptor associated factor, TRAF）家族成员是胞质接头蛋白，既行使接头蛋白的功能又具有泛素连接酶活性，可调控多种受体信号途径，进而激活核因子κB（nuclear factor kappa-B, NF-κB）、有丝分裂原活化蛋白激酶（mitogen-activated protein kinase, MAPK）和干扰素调节因子（interferon regulatory factor, IRF）信号。本研究旨在探讨TRAFs在不同组织和癌症类型中的表达以及在非小细胞肺癌（non-small cell lung cancer, NSCLC）中的mRNA表达、蛋白表达、预后意义及功能富集分析，为NSCLC的诊断和治疗提供新策略。

**方法:**

利用基因型组织表达数据库的RNA测序数据集分析TRAF家族成员在不同人体组织中的表达水平；利用癌细胞百科全书数据库中的RNA测序数据集分析TRAF家族成员在不同类型癌症细胞系中的表达水平；利用癌症基因组图谱（The Cancer Genome Atlas, TCGA）数据库的RNA测序数据集分析人类不同类型癌症中TRAF家族成员的mRNA表达水平；利用人类蛋白质图谱数据库中的免疫组化数据分析NSCLC包括肺腺癌和肺鳞癌中的TRAF家族成员的蛋白表达水平；利用Kaplan-Meier Plotter中的NSCLC患者的生存数据集，通过对数秩检验评估TRAF家族成员的表达与预后生存的相关性；利用TCGA数据库中NSCLC患者的RNA测序数据集对TRAF家族成员相关基因进行基因本体论功能注释分析和京都基因与基因组百科全书通路富集分析；利用基于TCGA数据库的RNA测序数据集，通过ESTIMATE对TRAF家族成员的表达水平与肿瘤免疫微环境进行相关性分析。

**结果:**

TRAF家族成员呈现显著的组织表达特异性，TRAF2、TRAF3、TRAF6和TRAF7广泛表达于多种组织，而TRAF1、TRAF4和TRAF5则在个别组织中表达受限；TRAF家族成员的表达在不同类型癌症细胞系中具有高度特异性；在肺腺癌和肺鳞癌组织的mRNA数据中，TRAF2、TRAF4、TRAF5和TRAF7的表达均上调，TRAF6的表达均下调，TRAF1仅在肺腺癌中上调，而TRAF3仅在肺鳞癌中上调；除TRAF3、TRAF4和TRAF7外，其他TRAF蛋白在肺腺癌和肺鳞癌组织中的免疫组化染色显著加深；TRAF2、TRAF4和TRAF7的表达与肺癌患者的总生存期呈负相关，TRAF3、TRAF5和TRAF6的表达与肺癌患者的总生存期呈正相关，而TRAF1的表达与肺癌患者的总生存期无明显相关性；TRAF家族成员差异性调控NF-κB、免疫应答、细胞黏附及RNA剪接相关的等多条信号通路；TRAF家族成员的表达水平与肿瘤免疫微环境中的免疫细胞浸润和基质细胞含量密切相关，且不同成员之间的相关性表现出正负差异。

**结论:**

TRAF家族成员在不同组织和癌症类型中表现出特异性差异表达，NSCLC中多数TRAF家族成员在mRNA和蛋白水平表达上调，其中，高表达的TRAF2、TRAF4和TRAF7提示预后不良，TRAF家族成员参与炎症、免疫、黏附及代谢等多条通路，并影响肿瘤免疫微环境。

## Abstract (en)

**Background and objective:**

Currently, lung cancer is one of the malignant tumors with a high morbidity and mortality all over the world. However, the exact mechanisms underlying lung cancer progression remain unclear. The tumor necrosis factor receptor associated factor (TRAF) family members are cytoplasmic adaptor proteins, which function as both adaptor proteins and ubiquitin ligases to regulate diverse receptor signalings, leading to the activation of nuclear factor kappa-B (NF-κB), mitogen-activated protein kinase (MAPK) and interferon regulatory factor (IRF) signaling. The aim of this study was to investigate the expression of TRAFs in different tissues and cancer types, as well as its mRNA expression, protein expression, prognostic significance and functional enrichment analysis in non-small cell lung cancer (NSCLC), in order to provide new strategies for the diagnosis and treatment of NSCLC.

**Methods:**

RNA sequencing data from the The Genotype-Tissue Expression database was used to analyze the expression patterns of TRAF family members in different human tissues. RNA sequencing data from the Cancer Cell Line Encyclopedia database was used to analyze the expression patterns of TRAF family members in different types of cancer cell lines. RNA sequencing data from the The Cancer Genome Atlas (TCGA) database was used to analyze the mRNA levels of TRAF family members across different types of human cancers. Immunohistochemistry (IHC) analyses from HPA database were used to analyze the TRAF protein levels in NSCLC [lung adenocarcinoma (LUAD) and lung squamous carcinoma (LUSC)]. Overall survival analysis was performed by Log-rank test using original data from Kaplan-Meier Plotter database to evaluate the correlation between TRAF expressions and prognosis. Gene Ontology and Kyoto Encyclopedia of Genes and Genomes pathway enrichment analyses were performed on the TRAF family-related genes using RNA sequencing data from the TCGA database for NSCLC. The correlation between the expression levels of TRAF family members and the tumor immune microenvironment was analyzed using the ESTIMATE algorithm based on RNA sequencing data from the TCGA database.

**Results:**

The TRAF family members exhibited significant tissue-specific expression heterogeneity. TRAF2, TRAF3, TRAF6 and TRAF7 were widely expressed in most tissues, while the expressions of TRAF1, TRAF4 and TRAF5 were restricted to specific tissues. The expressions of TRAF family members were highly specific among different types of cancer cell lines. In mRNA database of LUAD and LUSC, the expressions of TRAF2, TRAF4, TRAF5 and TRAF7 were significantly upregulated; while TRAF6 did the opposite; moveover, TRAF1 and TRAF3 only displayed a significant upregulation in LUAD and LUSC, respectively. Except for TRAF3, TRAF4 and TRAF7, other TRAF proteins displayed an obviously deeper IHC staining in LUAD and LUSC tissues compared with normal tissues. Additionally, patients with higher expression levels of TRAF2, TRAF4 and TRAF7 had shorter overall survival; while patients with higher expression levels of TRAF3, TRAF5 and TRAF6 had significantly longer overall survival; however, no significant difference had been observed between TRAF1 expression and the overall survival. TRAF family members differentially regulated multiple pathways, including NF-κB, immune response, cell adhesion and RNA splicing. The expression levels of TRAF family members were closely associated with immune cell infiltration and stromal cell content in the tumor immune microenvironment, with varying positive and negative correlations among different members.

**Conclusion:**

TRAF family members exhibit highly specific expression differences across different tissues and cancer types. Most TRAF proteins exhibit upregulation at both mRNA and protein levels in NSCLC, whereas, only upregulated expressions of TRAF2, TRAF4 and TRAF7 predict worse prognosis. The TRAF family members regulate processes such as inflammation, immunity, adhesion and splicing, and influence the tumor immune microenvironment.

肺癌是全球第二常见的原发性恶性肿瘤，是一种侵袭性极强的恶性肿瘤。其中，非小细胞肺癌（non-small cell lung cancer, NSCLC）是肺癌中最常见的病理类型，约80%的肺癌都是NSCLC^[1]^。针对NSCLC，医务人员在早期筛查、诊断、手术、放疗、化疗、分子靶向治疗和免疫治疗等方面做出了巨大的努力。但由于NSCLC极高的发病率和死亡率及较差的预后，其死亡人数在全球癌症相关疾病中位居前列^[2]^。因此，研究NSCLC恶性进展的分子机制，寻找与NSCLC发生发展相关的驱动基因至关重要。

肿瘤坏死因子（tumor necrosis factor, TNF）超家族由一组分泌型或膜结合型配体组成，而TNF受体（TNF receptor, TNFR）超家族则由其受体组成。这些受体发挥多种广泛的生理功能，包括发育、分化、细胞凋亡和免疫系统调节^[3]^。TNFR相关因子（TNFR associated factor, TRAF）蛋白便是这类胞内转接蛋白，可直接与细胞表面受体的胞内区结构域结合，介导胞内信号级联的激活^[4]^。迄今为止，已经在哺乳动物体内发现了7种不同的TRAF蛋白（TRAF1-7）^[5]^。

TRAFs是一个由细胞因子介导信号传递的接头蛋白家族，有一个相对保守的结构域，是多种细胞表面受体的接头蛋白，可调节多种胞内反应^[6]^。除TRAF7的C端为酪氨酸-天冬氨酸基元（Trp-Asp 40, WD40）重复序列外，其他TRAF蛋白的C端都有保守的TRAF结构域。TRAF结构域又分为两部分：TRAF-N结构域和TRAF-C结构域。TRAF-N区域有助于TRAF蛋白的寡聚化。TRAF-C区域不仅介导寡聚化，还介导与TNFR蛋白及其他胞质因子的相互作用^[7]^。除了TRAF1，其他所有TRAF蛋白的N端都含有一个保守的环指结构域和多个相邻的锌指结构域^[8]^。

TRAF蛋白通过多种信号途径，参与调控重要生物学过程，如细胞增殖、分化、存活和应激反应等^[9,10]^。TRAF1和TRAF2可调控激活核因子κB（nuclear factor kappa-B, NF-κB）和c-Jun氨基末端激酶（c-Jun N-terminal kinase, JNK）通路，但缺陷表型差异显著：TRAF1缺陷导致动脉粥样硬化、TNF高敏和T细胞功能障碍；而TRAF2缺陷则引起早夭、严重侏儒症、TNF高敏和干扰素无应答。TRAF3可调控视黄酸诱导基因I样受体（retinoic-acid inducible gene I-like receptor, RLR）和Toll样受体（Toll-like receptor, TLR）通路，缺陷表现为围产期及新生儿存活率低、严重侏儒、免疫器官萎缩伴T细胞功能障碍和外周血白细胞减少。TRAF4缺陷导致围产期存活率下降、呼吸系统畸形及神经管-骨骼发育异常，可能与转化生长因子-β（transforming growth factor-β, TGF-β）通路失调有关。TRAF5通过NF-κB及分化簇27（cluster of differentiation 27, CD27）和CD40通路调控B细胞应答、辅助T细胞1型/2型（helper T cell 1/2, Th1/Th2）分化和淋巴细胞活化。TRAF6作为多通路枢纽，激活NF-κB/JNK/p38并调控CD40/白细胞介素-1（interleukin-1, IL-1）/脂多糖（lipopolysaccharide, LPS）信号，其缺陷引发早夭、骨硬化症、牙发育异常、脾淋巴结肿大及系统性炎症反应，伴IL-1/LPS信号缺陷。TRAF7兼具有丝裂原活化蛋白激酶（mitogen-activated protein kinase, MAPK）激活、TLR调控及NF-κB抑制功能，目前尚未见基因缺陷表型报道^[5,11,12]^。

本研究将利用多个公共数据库的高通量数据，分析TRAF家族蛋白在正常组织和肺癌患者组织及细胞系中的表达，并对其相关基因进行基因本体论（Gene Ontology, GO）功能注释分析和京都基因与基因组百科全书（Kyoto Encyclopedia of Genes and Genomes, KEGG）通路富集分析，评估TRAF家族蛋白与肺癌患者生存预后及免疫微环境的相关性，为NSCLC的诊疗提供新策略和新靶点。

## 1 材料与方法

### 1.1 基因型组织表达数据库（The Genotype-Tissue Expression, GTEx）数据的收集和分析

GTEx数据库是一个庞大的基因表达数据集，它提供了人体不同组织中基因表达水平的全面信息。本研究基于GTEx数据库的RNA测序数据，涵盖多组织样本转录组信息，数据经每百万次读数（counts per million, CPM）标准化和对数转换以优化分布。采用R软件（v4.0.0+）进行差异表达分析及可视化，使用ggplot2、ggprism和ggpubr数据包构建图表。TRAF家族基因的组织间表达差异通过One-way ANOVA进行评估。

### 1.2 癌细胞百科全书数据库（Cancer Cell Line Encyclopedia, CCLE）数据的收集和分析

本研究利用CCLE数据库中的RNA测序数据分析TRAF家族在不同类型癌症细胞系中的表达模式。从CCLE数据库下载基因表达矩阵和样本注释信息，将基因表达数据与样本注释信息通过癌症依赖性图谱（The Cancer Dependency Map, DepMap）数据库的ID进行配对，并按照癌症类型进行分组。所有癌症类型按照基因表达中位数进行排序。以上分析采用R软件进行（v4.0.0+以上），并用tidyverse和ggplot2数据包进行可视化及统计分析。

### 1.3 癌症基因组图谱（The Cancer Genome Atlas, TCGA）数据的收集与分析

TCGA数据库收录了33种癌症类型中超过20,000种原发性癌症组织样本的测序数据。本研究通过TCGA数据库中每百万次转录本（transcripts per million, TPM）格式的RNA测序数据分析，将不同类型肿瘤组织与正常组织作比较，分析TRAF家族成员在人多种类型恶性肿瘤中的mRNA表达差异。用于分析的目标分子靶点包括：TRAF1（ENSG00000056558.11）、TRAF2（ENSG00000056555.13）、TRAF3（ENSG00000072778.12）、TRAF4（ENSG00000183476.9）、TRAF5（ENSG00000005075.12）、TRAF6（ENSG00000175104.14）及TRAF7（ENSG00000168313.15）。数据进行对数变换后，通过Wilcoxon秩和检验分析统计学差异。同时，为了筛选TRAF家族蛋白参与细胞功能调控的主要过程和主要通路，我们计算了TRAF家族成员与全基因组基因的Spearman相关系数，并筛选与每个TRAF基因显著相关的基因集（|ρ|>0.4，校正后P<0.05，Benjaminiand-Hochberg法校正），随后对这些基因集分别进行GO功能注释分析和KEGG通路富集分析。GO分析包含分子功能（molecular function, MF）、生物过程（biological process, BP）和细胞组分（cellular component, CC）三个层面。富集采用超几何检验。以上分析采用R软件进行（v4.2.1），并用ggplot2、stats、car和clusterProfiler数据包进行可视化及统计分析。

### 1.4 人类蛋白质图谱数据库（The Human Protein Atlas, HPA）数据的收集与分析

HPA数据库中使用了大量的高质量抗体及免疫组织化学（immunohistochemistry, IHC）技术来检测和定位人类蛋白质在组织、细胞和器官中的表达。本研究通过HPA数据库分析，将肺腺癌（lung adenocarcinoma, LUAD）组织、肺鳞癌（lung squamous carcinoma, LUSC）组织与正常组织作比较，分析TRAF家族成员在人多种类型恶性肿瘤中的蛋白表达差异。

### 1.5 卡普兰-梅尔绘图者数据库（Kaplan-Meier Plotter, KM plotter）数据的收集与分析

KM plotter数据库是一种基于生物标志物评估的在线荟萃分析工具，它能基于大型样本数据集来评估不同生物标志物的表达对不同类型癌症患者生存的影响。Kaplan-Meier生存分析则通常用于估计特定时间点上的生存率，评估单一因素对生存期的影响。本研究通过KM plotter数据库分析TRAF1-7的表达高低对肺癌患者总生存期（overall survival, OS）的影响，将P<0.05视为差异具有统计学意义。

### 1.6 TRAFs表达与肿瘤免疫微环境的相关性分析

本研究从TCGA数据库获取标准化的基因表达数据，采用肿瘤免疫评估法（Estimation of STromal and Immune cells in MAlignant Tumor tissues using Expression data, ESTIMATE）评估每个样本的免疫细胞浸润水平（免疫评分）和基质细胞含量（基质评分），数据预处理涵盖标准化及缺失值剔除，并使用Spearman等级相关分析评估TRAF家族成员的表达水平与免疫评分和基质评分之间的相关性，计算相关系数（ρ）和统计显著性（P值）。采用R软件（v4.4.0）进行数据处理和统计分析，使用ggplot2数据包绘制散点图，并通过线性回归拟合展示表达量与评分之间的总体趋势。所有统计分析的显著性水平设定为P<0.05。

## 2 结果

### 2.1 TRAFs在不同人体组织中的表达水平分析

为了了解TRAFs在不同人体组织中的表达模式，使用R语言对GTEx数据库中的RNA测序数据进行了分析（附图1，http://www.lungca.org/files/2025s15_supp1.pdf）。结果显示，TRAF家族成员在各组织中的分布情况存在差异。TRAF2、TRAF3、TRAF6和TRAF7广泛表达于几乎所有组织和器官。相比之下，TRAF1在骨髓、肌肉和胰腺中的表达较低，TRAF4则在心脏和肌肉中表达较少，TRAF5在肝脏、心脏和肌肉中的表达较低。

### 2.2 TRAFs在不同类型癌症细胞系中的表达谱分析

为探讨TRAF家族成员在不同类型癌症细胞系中的表达模式，对CCLE数据库中的RNA数据进行了分析，不同TRAF基因在各类型癌症细胞系中存在显著的表达差异。TRAF1在淋巴瘤细胞中表达最高，TRAF2在肝癌细胞中表达最显著，TRAF3在肾上腺癌细胞中呈高表达，TRAF4在乳腺癌细胞表达最高，TRAF5在眼癌细胞中表达最显著，TRAF6在白血病中表达最显著，TRAF7在肾上腺癌细胞中表达水平较高（附图2，http://www.lungca.org/files/2025s15_supp2.pdf）。这些结果提示TRAF家族成员的表达在不同类型癌症细胞系中具有高度特异性，进一步分析其在癌组织与正常组织中的差异表达，将有助于揭示其在肿瘤发生中的潜在作用。

### 2.3 TRAFs在不同类型癌症组织中的mRNA表达变化

为了解TRAF家族成员在人类各类癌症中的表达情况，我们通过TCGA数据库中的RNA测序数据分析，将不同类型肿瘤组织与正常组织作比较，利用R语言分析TRAF家族成员在人多种类型恶性肿瘤中的mRNA表达差异。如图1所示，与正常组织相比，TRAF家族成员在大多数人类癌症中表达上调。特别地，TRAF1-7在某些类型的人类癌症中同时上调，其中包括胆管癌（cholangio carcinoma, CHOL）、肝细胞癌（liver hepatocellular carcinoma, LIHC）和胃腺癌（stomach adenocarcinoma, STAD）（P<0.001）。在LUAD和LUSC组织的mRNA数据中，TRAF2、TRAF4、TRAF5和TRAF7均表达上调（P<0.001），TRAF6表达下调（P<0.01），TRAF1仅在LUAD中表达上调（P<0.001），TRAF3仅在LUSC中表达上调（P<0.001），以上结果表明，TRAF家族成员在NSCLC中具有潜在的促癌功能。

**图1:**
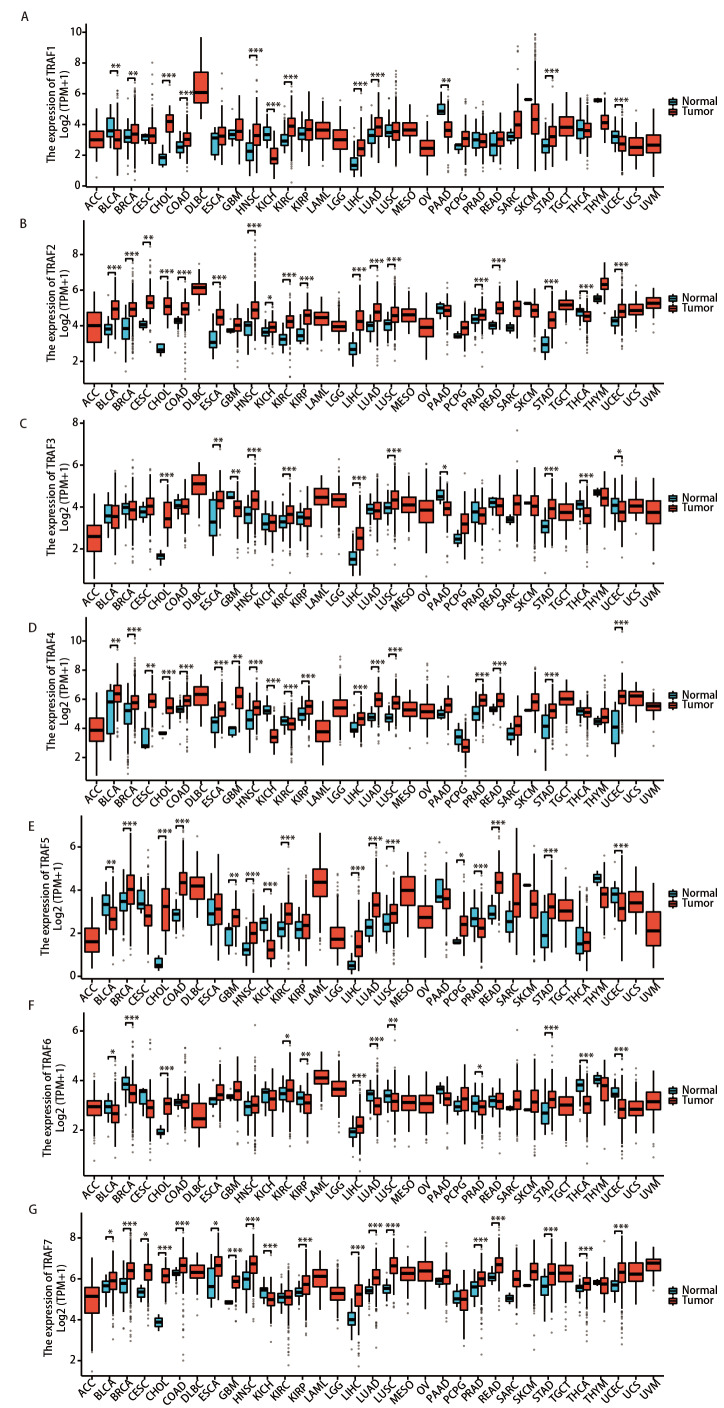
根据TCGA数据库的数据，分析TRAF1-7 mRNA 在多种人类癌症（包括NSCLC：LUAD和LUSC）中肿瘤组织与正常组织的表达情况。Y轴以log2(TPM+1)表示TRAF1-7的表达水平。蓝色箱体和红色箱体分别代表正常组织和肿瘤组织。A：TRAF1；B：TRAF2；C：TRAF3；D：TRAF4；E：TRAF5；F：TRAF6；G：TRAF7。*：P<0.05；**：P<0.01；***：P<0.001。

### 2.4 TRAFs在NSCLC中的蛋白表达变化

当涉及到基因表达时，生物体内的蛋白水平和mRNA水平往往不一致。接下来，利用HPA数据库分析了NSCLC（LUAD和LUSC）中的TRAF蛋白表达水平。如图2所示，利用该数据库高质量抗体的IHC分析，结果发现TRAF家族成员大多在NSCLC组织中上调。将LUAD组织、LUSC组织与正常组织作比较发现，除TRAF3、TRAF4和TRAF7外，其他TRAF蛋白在LUAD和LUSC组织中的IHC染色显著加深，表明在NSCLC组织中的表达增加。值得注意的是，TRAF6的蛋白上调与TRAF6的mRNA下调并不一致，这表明TRAF6可能发生转录后调控和翻译后修饰。综上，TRAF家族成员在mRNA水平和蛋白质水平的表达上调，表明其对NSCLC的恶性进展至关重要。

**图2:**
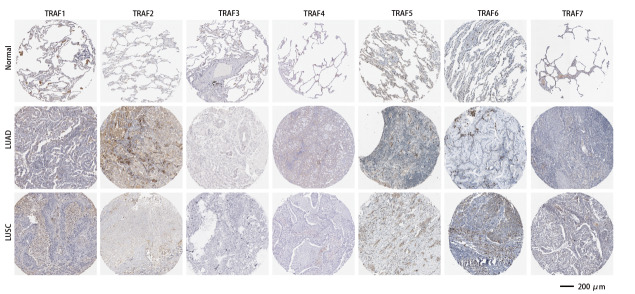
利用HPA数据库中的IHC分析TRAF1-7在正常肺组织和NSCLC组织（包括LUAD和LUSC）中的蛋白表达。

### 2.5 TRAFs在肺癌预后生存中的意义

在初步证实了TRAF家族成员的上调表达与NSCLC诊断的关联性后，进一步了解其上调是否与NSCLC的预后有关。我们利用KM Plotter数据库中的生存数据，通过对数秩检验进行了Kaplan-Meier生存率分析。如图3所示，TRAF2、TRAF4和TRAF7高表达患者的OS短于低表达患者（P<0.05）；TRAF3、TRAF5和TRAF6高表达患者的OS显著长于低表达患者（P<0.001）；而TRAF1高表达患者的OS与低表达患者之间无明显差异（P>0.06）。以上结果表明，TRAF2、TRAF4和TRAF7表达较高的患者预后较差；而TRAF3、TRAF5和TRAF6表达较高的患者预后较好。

**图3:**
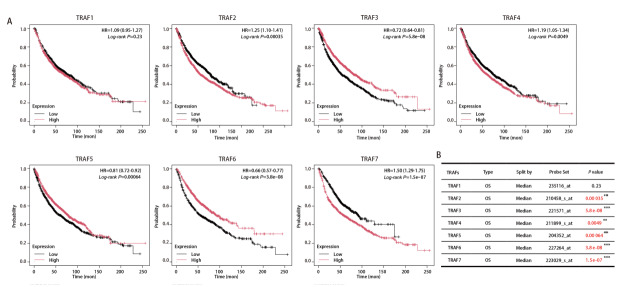
TRAF1-7在NSCLC患者中的预后生存意义。A：使用KM Plotter在线数据库生存分析工具对TCGA中的患者数据进行总生存期分析；B：生存分析中的各参数和P值。**：P<0.01；***：P<0.001。

### 2.6 TRAFs的GO功能注释分析和KEGG通路富集分析

TRAF家族成员在NSCLC中的表达差异与预后相关性差异均提示，该家族可能通过调控多条不同功能的通路参与NSCLC的发生发展。为进一步推断可能的调控机制，对TCGA数据库中NSCLC肿瘤组织的RNA测序数据进行GO功能注释分析和KEGG通路富集分析，结果如图4和图5所示，TRAF1主要富集于免疫相关通路，包括细胞因子信号传导、T细胞分化及白细胞介导的免疫相关过程。TRAF2主要关联神经退行性疾病，参与赖氨酸降解、转录调控及组蛋白甲基化等表观遗传修饰过程。TRAF3参与病毒应答、激素信号转导，同时涉及GTP酶信号传导、DNA修复和核质转运等基础细胞功能。TRAF4关联神经退行性疾病，富集于细胞维持过程相关通路，并参与RNA剪接、核糖核蛋白复合物生成及线粒体组织等RNA代谢相关过程。TRAF5主要参与RNA剪接调控，富集于剪接体复合物及细胞骨架相关过程。TRAF6介导细胞运输和信号转导，显著富集于内吞、自噬、囊泡运输及GTP酶信号传导等通路。TRAF7主要富集于神经退行性疾病和细菌感染相关通路，同时富集于核糖核蛋白复合物生成、RNA剪接及蛋白质定位等细胞过程。以上结果提示，TRAF家族成员在RNA剪接、免疫调控、细胞生长及代谢等生物学过程中具有广泛而重要的功能。

**图4:**
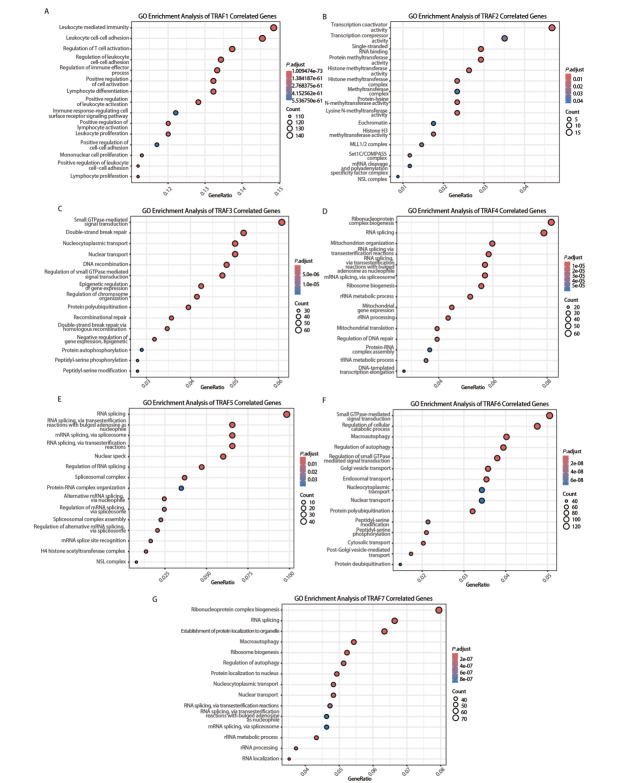
根据TCGA数据库中的RNA测序数据，对TRAF家族成员相关基因进行GO功能注释分析。A：TRAF1；B：TRAF2；C：TRAF3；D：TRAF4；E：TRAF5；F：TRAF6；G：TRAF7。

**图5:**
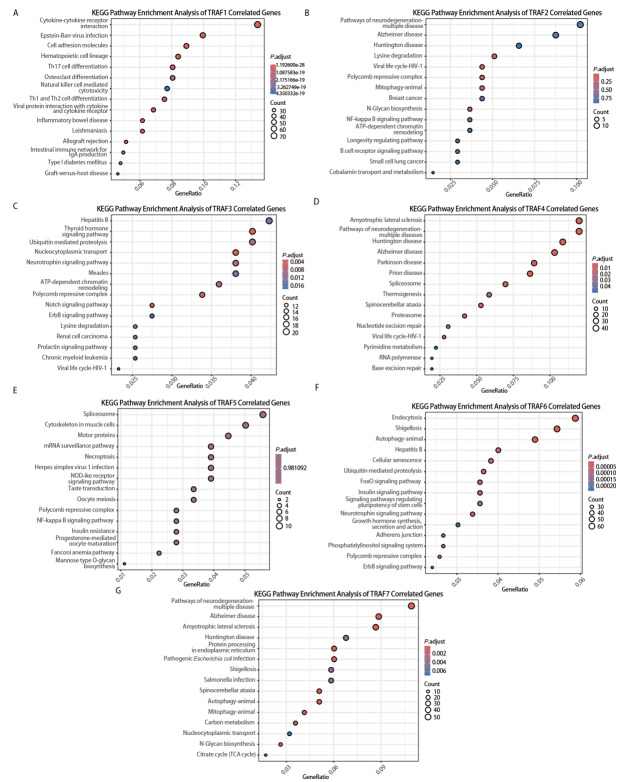
根据TCGA数据库中的RNA测序数据，对TRAFs家族成员相关基因进行KEGG通路富集分析。A：TRAF1；B：TRAF2；C：TRAF3；D：TRAF4；E：TRAF5；F：TRAF6；G：TRAF7。

### 2.7 TRAFs与肿瘤免疫微环境的相关性分析

在初步探讨了TRAF家族成员广泛参与免疫调节相关信号通路的基础上，我们进一步分析了其与肿瘤免疫微环境的关系，利用ESTIMATE算法分析TCGA数据库的RNA测序数据，以评估每个样本的免疫细胞浸润水平和基质细胞含量，并通过Spearman等级相关分析评估其与TRAF家族成员表达水平的相关性。如图6所示，TRAF1、TRAF3和TRAF5的表达与免疫评分及基质评分呈正相关，TRAF2、TRAF4和TRAF7的表达则与之呈负相关，TRAF6的表达与免疫评分的相关性不显著，但与基质评分呈正相关。以上结果表明，TRAF家族成员的表达与肿瘤免疫微环境密切相关。

**图6:**
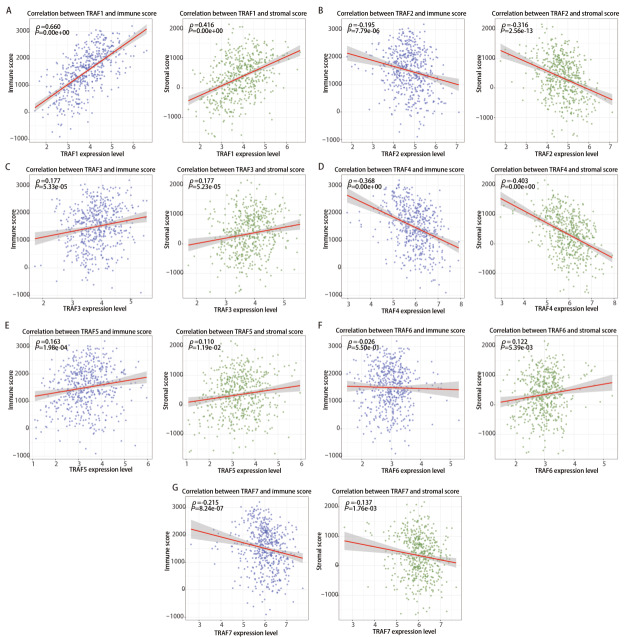
通过ESTIMATE算法对TRAFs家族成员的表达水平与肿瘤免疫微环境进行相关性分析。A：TRAF1；B：TRAF2；C：TRAF3；D：TRAF4；E：TRAF5；F：TRAF6；G：TRAF7。

## 3 讨论

肺癌是全球癌症相关死亡的主要原因，其死亡人数在全球癌症相关疾病中位居前列^[1]^。其中NSCLC是肺恶性肿瘤的主要病理类型之一，其恶性程度极高^[2]^。因此，对于肺癌恶性进展的机制研究十分迫切，这将为肺癌分子靶向治疗的靶点选择和策略优化提供重要线索。

有研究^[9,10]^表明，TRAFs参与多种的信号通路以调控重要生物过程，且许多TRAFs参与的信号途径与癌症发病机制密切关联。本研究系统分析了TRAFs在正常组织、不同类型癌症细胞系及NSCLC组织中的表达模式及其与预后的关系，探讨了TRAFs参与调控的信号通路，并评估了其与肿瘤免疫微环境的相关性。本研究发现，TRAFs在正常组织中呈现显著的组织特异性分布：TRAF2、TRAF3、TRAF6及TRAF7广泛表达于多种组织，而TRAF1、TRAF4和TRAF5则表现出组织特异性表达，提示其功能可能受微环境调控。不仅如此，在不同类型癌症细胞系中，TRAFs家族成员的表达也呈现高度癌种依赖性，如TRAF1在淋巴瘤、TRAF2在肝癌、TRAF4在乳腺癌及TRAF6在白血病中表达最高，暗示其可能作为特定癌症的潜在分子标志物。

值得注意的是，在NSCLC中，TRAF2和TRAF5的mRNA及蛋白水平均显著上调，且与患者预后密切相关。其中，TRAF2、TRAF4和TRAF7的高表达与生存期缩短有关，而TRAF3、TRAF5和TRAF6的高表达提示预后改善，其mRNA和蛋白水平的表达与患者预后并不完全一致，这更加提示TRAFs可能具有极其复杂的调控机制和多样的生物学功能。有研究^[13,14]^表明，TRAF1在NSCLC中显著上调，并与较差的OS相关，功能实验发现了其促进细胞增殖、侵袭、分化等方面的致癌功能。TRAF2参与细胞增殖、转移、血管生成和放疗抵抗等过程^[15⇓⇓-18]^。然而，TRAF3的作用存在争议，有研究^[19,20]^指出其在NSCLC中表达下调，抑制细胞增殖和迁移，也有研究^[21]^发现其表达上调并促进自噬以发挥致癌作用。TRAF4在NSCLC中的过表达已被多个研究小组广泛认可，有研究^[22⇓⇓-25]^显示其促进细胞增殖、侵袭、上皮间充质转化和放疗抵抗。2024年Zhou等^[26]^研究发现TRAF5在LUAD组织中表达显著降低，其低表达与预后不良相关，上调TRAF5的表达可抑制细胞活力、迁移和侵袭，并诱导凋亡。TRAF6被报道在NSCLC中表达上调，促进细胞增殖、侵袭、迁移和抗药性^[27⇓-29]^，但也有研究^[30]^报道其表达下调并抑制肿瘤进展，值得注意的是，TRAF6的蛋白水平升高与mRNA表达下调形成显著矛盾，暗示其可能通过泛素化或磷酸化等转录后调控和翻译后修饰机制参与功能调控，这为解析TRAF6在肿瘤中的功能争议提供了新视角。目前，关于TRAF7在NSCLC中的作用尚缺乏报道，需要在未来进一步探索，但是结合TRAF7区别于其他家族成员的C端结构，及其在NSCLC中的表达上调、与患者预后以及相关的功能富集分析和肿瘤免疫微环境的相关性，我们有理由相信，TRAF7极有可能是NSCLC治疗干预的潜在靶点。

综上所述，TRAFs家族成员在NSCLC的发生、发展及转移过程中发挥了重要作用，主要通过影响细胞存活、增殖、侵袭、迁移、炎症反应、放射抗性及耐药性等多个方面来调控肺癌的恶性进展。尽管目前对TRAFs蛋白如何影响NSCLC的具体机制仍未完全阐明，但越来越多的研究表明，它们可能成为肺癌治疗的新靶点，并为探索新的治疗策略提供了重要依据。

然而，本研究仍然存在一定的局限性。首先，TRAFs家族成员的表达调控及其具体功能机制仍需进一步深入探讨，尤其是TRAF6的转录后调控和翻译后修饰在NSCLC中的作用尚不明确，该调控和修饰如何影响下游信号通路仍有待研究。其次，本研究主要依赖公共数据库进行回顾性分析，尽管这些数据为研究提供了重要参考，但可能存在批次效应、数据偏倚及样本代表性不足等问题，会影响分析结果的可靠性：例如，TRAF2和TRAF5生存分析的部分曲线中存在少量交叉，这表明高低表达组间在不同时间段内的生存率有所差异。这可能是由于公共数据库的数据局限性造成的，如样本量过小或某一组阳性例数过少等，也可能存在其他混杂因素，如性别和年龄等。因此，后续研究需要结合前瞻性临床队列研究，并开展多变量分析，以提高数据的准确性和临床相关性。最后，仍需体内外功能实验来进一步验证TRAFs家族成员在NSCLC发生发展中的具体功能，以明确其作为治疗靶点的可行性和有效性。未来，通过更精准的分子机制研究和临床验证，有望进一步挖掘TRAFs家族蛋白在NSCLC中的潜在应用价值，为个性化治疗提供新的思路。
